# Emergency treatment on facial laceration of dog bite wounds with immediate primary closure: a prospective randomized trial study

**DOI:** 10.1186/1471-227X-13-S1-S2

**Published:** 2013-07-04

**Authors:** Chen Rui-feng, Huang Li-song, Zheng Ji-bo, Wang Li-qiu

**Affiliations:** 1 Department of Emergency, Naval General Hospital, Beijing 100048, China

## Abstract

**Background:**

To investigate the emergency treatment on facial laceration of dog bite wounds and identify whether immediate primary closure is feasible.

**Methods:**

Six hundred cases with facial laceration attacked by dog were divided into two groups randomly and evenly. After thorough debridement, the facial lacerations of group A were left open, while the lacerations of group B were undertaken immediate primary closure. Antibiotics use was administrated only after wound infected, not prophylactically given. The infection rate, infection time and healing time were analyzed.

**Results:**

The infection rate of group A and B was 8.3% and 6.3% respectively (*P*>0.05); the infection time was 26.3±11.6h and 24.9±13.8h respectively (P>0.05), the healing time was 9.12±1.30d and 6.57±0.49d respectively (P<0.05) in taintless cases, 14.24±2.63d and 10.65±1.69d respectively (P<0.05) in infected cases.

Compared with group A, there was no evident tendency in increasing infection rate (8.3% in group A and 6.3% in group B respectively) and infection period (26.3±11.6h in group A and 24.9±13.8h in group B respectively) in group B. Meanwhile, in group B, the wound healing time was shorter than group A statistically in both taintless cases (9.12±1.30d in group A and 6.57±0.49d in group B respectively) and infected cases (14.24±2.63d in group A and 10.65±1.69d in group B respectively).

**Conclusion:**

The facial laceration of dog bite wounds should be primary closed immediately after formal and thoroughly debridement. And the primary closure would shorten the healing time of the dog bite wounds without increasing the rate and period of infection. There is no potentiality of increasing infection incidence and infection speed, compared immediate primary closure with the wounds left open. On the contrary, primary closure the wounds can promote its primary healing. Prophylactic antibiotics administration was not recommended. and the important facial organ or tissue injuries should be secondary reconditioned.

## Background

In recent years, more and more people suffered from dog bite, along with the increasing amount of domesticated dogs. According to the data from the Centers for Disease Control and Prevention (CDC) of Beijing, there was over 100,000 people were attacked by dogs in 2007 in Beijing, which exceeded over 180,000 in 2011. About 10% of dog bite cases were facial wounds. As a special type of wound, dog bite wound had its characters, such as high infection rate and serious complications. The local infection, sometimes even intracranial infection, of the facial dog bite wound was inevitable and unmanageable generally. Although some pertinent literature have been published about dog bite facial wound, prospective studies was rarely concerned. At present, controversial focus existed about facial dog bite laceration management: One is whether it’s appropriate to perform immediate primary closure; another is whether it is essential to give prophylactic antibiotic. In order to get a definite answer about these topics, we carried out this prospective trial study.

## Patients and methods

Patients of all ages and gender attending Rabies Prophylaxis and Immunity Clinic of Beijing with facial dog bite were enrolled in the prospective, randomized trial. The facial lacerated wounds requiring surgical treatment (more than 2cm) were entered into the trial. Punture wounds (less than 2mm), small laceration (less than 2cm), infected wounds at presentation or visited doctor’s office after injured more than 8h, wounds with skin loss requiring plastic surgery, or patients with immune deficiency, using immunosuppressive agent, autoimmune disorder and diabetes were excluded. All the patients were randomized by block randomization and distributed to control group A and trail group B by block random digits table. The therapeutic schedules were explained to the patients in each group, and the consent form was signed. The patients who refused the therapeutic strategy were excluded from the trial.

All the facial lacerations underwent thorough debridement as below.

### Cleaning and disinfection

In order to release the pain of patients, local anesthetic was administrated before wounds cleaning. After covering with sterilized dressing to the wounds, aseptic carbasus was used to scrub the area around the wounds 2-3 times with 20% liquid soap and water. Subsequently, the wounds were alternating douched with 20% liquid soap and physiological saline, and then 3% hydrogen peroxide and physiological saline. The total cleaning time was not less than 15 minutes each wound. A great quantity of 0.05% iso-osmia iodophors (1 portion 0.5% iodophors stock solution + 9 portion physiological saline) was used to disinfect the wounds, not less than 5 minutes. Caution, during the whole cleaning and disinfection procedure, the interior part of the wounds was more important than the surface of the wounds.

### Debridement

All the inactivated tissues, coagulated blood, foreign material and serious contaminated tissues were carefully removed to expose surrounding healthy tissue. It was essential to remain their integrity as far as possible, so as to be repaired afterwards. The last procedure of debridement was douched the inside part of laceration with 0.05% iodophors again, the sterile gloves, aseptic covers and surgical instruments was prepared for tissue repair. At this time, passive immunity, if necessary, should be given (Rabies Immunoglobulin or Rabies Antiserm). Regarding the importance of impaired facial organ or tissue, it was essential to remain their integrity which could be repaired afterwards.

### Important tissue repair

All the important impaired or missing facial organ or tissues (such as eyelid, eyeball, nasolacrimal canal, parotid, nose, ear etc) were repaired with a suitable operation after the lacerations reached clinical healing.

### Wound closure

After thorough cleaning and debridement, the laceration was left open in group A; while those in group B was closed immediately. The 5/0 or 6/0 stylolite was used.

All the patients were administrated rabies prophylactic active immunity and/or passive immunity according to *Rabies Exposure Prophylactic and Handle Working Standard* (*2009 edition*)*.* Tetanus antitoxin (TAT) was given, if necessary. Drainage was carried out as the actual condition of laceration. Drain was placed innermost of the wound and replaced or pulled out according to the drainage quantity, usually 24h-48h after operation. All the wounds were covered with sterilized dressing and changed dressings 24h-48h after operation. The stitches in group B was removed 5d-7d after operation according to the wound healing condition. Antibiotic was used only after the wounds infection taking place.

## Oberservation index

### Infection rate

The criterion of infection should met one of three major criterias: fever( body temperature ≥ 38°C), abscess, and lymphangitis; or four of five minor criterias: wound-associated erythema that extended more than 3cm from the edge of the wound, tenderness at the wound site, swelling at the site, purulent drainage, and white-cell count in the peripheral blood 12,000 /ml.

### Infection time

The interval from being bitten to emerging infection indication (calculated the time in hours).

### Recovery time

The interval from being bitten to the wounds arriving clinical healing (calculated the time in days).

## Statistical analysis

Statistical analysis was carried out with SPSS 13.0 to compare the two groups. The Chi square test and t-test was applied. Statistical significance was set at α=0.05.

## Results

Between January 2006 and December 2011, 600 patients entered in this study: 272 male and 328 female. The age range was 1-64 years with 53% of the patients less than 10 years old. The average length of the wounds was 3.15±0.27cm, and the average wound amount was 3.6±1.8. Some patients were lost or serious damaged their organs by dog bite: 5 cases lost eyeballs, 7 thoroughly lost their ears, 12 lost a part of ears, 15 lost a part of noses, 21 parotid glands were damaged, 13 nasolacrimal canals were torn and 33 eyelids were lacerated. (A facial dog bite case seen in graph 1 and 2)

**Figure 1:**
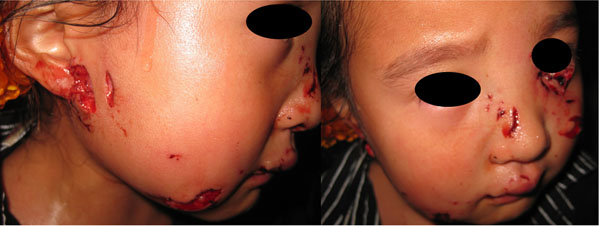
Little girl bitten by a dog. Her left nasolacrimal canal and eyelids were torn, and her right parotid gland and nose were also injured.

**Figure 2:**
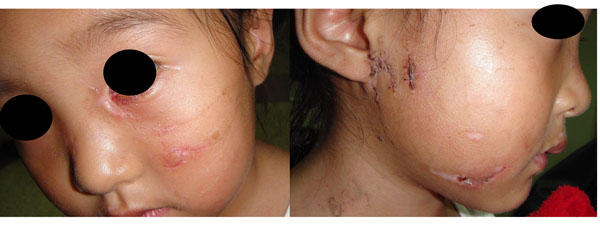
14d after bite. We carried out immediate primary closure and restored the eyelids and parotid gland immediately. The stitches were removed on the 5d, and then reconditioned the nasolacrimal canal.

After randomization, 129 male(43.0%) and 171 female(57.0%) entered control group(average age: 27.27±10.07 years old); 143 male(47.3%) and 157 female(52.3%) entered trial group(average age: 25.79±12.38 years old).

None of the enrolled patients fell rabies and intracranial infection. The wound infection rate of the two groups (A and B) was 8.3% and 6.3% respectively (P>0.05). The infection time of the two groups was 26.3±11.6h and 24.9±13.8h respectively(P>0.05). The recovery time in infection patients of the two groups was 9.12±1.30d and 6.57±0.49 d respectively (P<0.05), and in taintless patients of the two groups was 14.24±2.63 d and 10.65±1.69 d respectively (P<0.05). (Table 1)

**Table 1:** The results of two groups after surgery

Group	Infection rate	Infection	Recover time(d)	
		time(h)	infection	taintless
**Control group(A)**	8.3%(25/300)	26.3±11.6	9.12±1.30	14.24±2.63

**Trail group(B)**	6.3%(19/300)	24.9±13.8	6.57±0.49*	10.65±1.69*

* compared with control group, P<0.05

## Discussion

Dog bite wound is a special surgical wound. High infection rate (range from 18% to 25%), serious complications, and almost 100% fatality rate of rabies was reported [1,2]. During seven years from the beginning of R*abies Prophylaxis and Immunity Clinic* established, more than 50,000 dog bite patients had visited the clinic, among which the facial dog bite patients occupied 13.4%. The facial bite wounds could not only induce severe complications, such as fatal intracranial infection, fistula of parotid gland, ectropion, and nasolacrimal canal injury, but also resulted in facial cicatrix which affected facial cosmetology. As far as contaminated with the oral flora of the culprit concerned, the bite wounds infection prevention should also be concerned seriously, except for rabies and tetanus prophylaxis. Furthermore, in the management of non-complicated bite wounds, primary closure and prophylactic antibiotics application in initially uninfected wounds were still controversial issues [3-6].

## Whether immediate primary closure increased infection rate

Our findings suggested that immediate primary closure had no statistical discrepancy compared with the dog bite facial wounds left open in infection rate and infection time. In other words, immediate primary closure the facial dog bite lacerations neither increase the wounds infection rate nor accelerate wounds infection.

However, there was a very important issue should be emphasized, that is primary closure must be enforced after thorough cleaning, disinfection and debridement. Our previous study had indicated that using 0.05% iodophors instead of 2.5%-3.5% iodine tincture and 75% alcohol to sterilize the inside of the dog bite wounds could decrease the infection rate to 10% approximately without prophylactic antibiotics (facial wound is about 7.5%) [7]. Although, in the past 7 years over 50,000 dog bite patients visited to our clinic, some of which bitten by certified rabies dogs, none of the patients had acquired rabies. Our study had suggested that thorough wounds debridement, normal passive immunity and active immunity were the most valid intervention to prevent rabies [7-9]. Therefore, we believed that thorough debridement without delay was not only one of the key points in preventing rabies but also in decreasing wound infection rate. And we enforced immediate primary closure to dog bite facial laceration.

## The effect of primary closure to facial laceration healing

It was obvious in our study that immediate primary closure had great promotion in facial laceration healing. The healing time of taintless patients in trial group and control group was 6.57d and 9.12d respectively (P<0.05), while of in infected patients was 10.65d and 14.24d (P<0.05) respectively. It is well known that debridement is designed to make contaminated wound into clean wound, so that it can be sutured immediately and reach primary healing. Because if the wound is left open, it would get secondary healing (scar healing), and the wound would experience inflammation-hyperplasia of granulation tissue formation-scar formation in the process. The healing time will be extended, and the function would not recovery completely due to scar hyperplasia or contracture [3-6]. This was confirmed by our findings. Furthermore, in our clinical work we found that secondary healing was more poor than primary healing on scar size and appearance looking. Especially involved the eyes, nose, ears and mouth, the scar of the lacerations could induce serious deformity or complications (such as ectropion and trichiasis). Even in those patients with scar diathesis, the scar would more obvious and outstanding which could bring great physical and psychological harm to them. So, we recommend primary closure to suitable facial dog bite laceration after thorough debridement in order to reach primary healing.

## Antibiotics use

Whether prophylactic antibiotics using routinely is a controversial issue on faial dog bite laceration [1,2]. Our study suggested that without using antibiotics to prevent infection, the infection rate of facial dog bite laceration was about 8.3%. We believe that it was not necessarily to use antibiotics preventively. The infection rate of our study had large different with some documents. And we considered the surgery debridement method was the main factor in anti-infection.

It had reported that infection type of the dog bite wounds included aerobic and anaerobic infection. Canis pasteurella species is the most common stain, Streptococcus, Staphylococci, Moraella and Neisseria is the most common aerobic, and Fusobacterium, Bacteroides and Porohyromonas is the most common anaerobic. Furthermore, most species isolated from infected bite wounds are β-lactamase producers [1,2,11,12]. Considering the type of bacterias and sensitive antibiotics, the author recommend a combination of β-lactam antibiotic and aβ-lactamase inhibitor, a second-generation cephalosporin or clindamycin and a fluorquinolone, in antibiotics administration.

## Important facial organ restoration

Facial dog bite not only could induce serious soft tissue injuries but also can induce important organ, and tissue loss, such as eyeballs, eyelids, nasolarimal canal, parotid, ears, nose and lips, and resulted in serious complications and consequences (physiological and psychological trauma). Although the time of the injuried organ restoration was disputed, the author considered that the serious laceration and relavant blood vessel, nerve injury had negative influence in the time of organ restoration. And it was very important to check and protect those injured organs in the primary treatment to avoid secondary injuries.

## Limitations

It took us 6 years and a lot of effort to accomplish the prospective randomized controlled trial study. Although we have finally achieved the anticipated results, there were still some limitations in this study. Owning to the financial and laboratory conditions, we had not carried out the bacterial culture and the medicine sensitive experiments of the infected wounds. So we had to use antibiotics empirically based on previous literature reports.

## Conclusion

Our study showed that facial laceration of dog bite wounds should be immediately primarily closed after formal and thorough debridement. The cleaning, disinfection and debridement to the wounds was very important for bacterial and rabies virus infection prevention. There is no potentiality of increasing infection incidence and infection speed, compared immediate primary closure with the wounds left open. On the contrary, primary closure the wounds can promote its primary healing. Prophylactic antibiotics administration was not recommended and the important facial organ or tissue injuries should be secondary reconditioned.

## Competing interests

All of the authors declared that they had no competing interests.

## Authors’ contributions

CR-f was fully in charge of the study, involved in the design, demonstration, implementation, data collection and statistical analysis, and manuscript composition. WL-q was involved in the design, surveillance and manuscript revising for important intellectual content. HL-s was involved in writing the manuscript. HL-s and ZJ-b were coordinated the data collection and implementation. All the authors read and approved the final manuscript.

## Declarations

This article has been published as part of *BMC Emergency Medicine* Volume 13 Supplement 1, 2013: Proceedings of the 2012 Emergency Medicine Annual Congress. The full contents of the supplement are available online at http://www.biomedcentral.com/bmcemergmed/supplements/13/S1.

The publication costs for this article was funded by Naval General Hospital, Beijing 100048, China.

## References

[B1] Talan DA Citron DM Abrahamian FM Moran GJ Goldstein EJ Bacteriologic analysis of infected dog and cat bites. Emergency Medicine Animal Bite Infection Study Group N Engl J Med 1999 340 85 92 10.1056/NEJM199901143400202 9887159

[B2] Taplitz RA Managing bite wounds. Currently recommended antibiotics for treatment and prophylaxis Postgrad Med 2004 116 49 52 15323154 10.3810/pgm.2004.08.1572

[B3] Maimaris C Quinton DN Dog-bite lacerations: a controlled trial of primary wound closure Archives of emergency medicine 1988 5 156 161 10.1136/emj.5.3.156 3178974 PMC1285519

[B4] Capellan O Hollander JE Management of lacerations in the emergency department Emerg Med Clin N Am 2003 21 205 231 10.1016/s0733-8627(02)00087-1 12630738

[B5] Garbutt F Jenner R Wound closure in animal bites Emerg Med J 2004 21 589 590 15333543 10.1136/emj.2004.017962 PMC1726453

[B6] Smith MR Walker A Brenchley J Barking up the wrong tree? A survey of dog bite wound management Emerg Med J 2003 20 253 255 10.1136/emj.20.3.253 12748142 PMC1726116

[B7] Rui-feng CHEN Li-qiu WANG Li-song HUANG Study: debridement of dog bite wounds China journal of emergency resuscitation and disaster medicine 2010 5 23 27

[B8] Rui-feng CHEN Li-qiu WANG Li-song HUANG Distribution and characteristics of infection in dog bite wounds China journal of emergency resuscitation and disaster medicine 2011 6 21 23

[B9] Rui-feng CHEN Li-qiu WANG The effect analysis of debridement and immunifaction after rabid dog bite Guideof China medicine 2012 5 401 402

[B10] Stefanopoulos PK Tarantzopoulou AD Facial bite wounds: management update Int. J. Oral. Maxillofac. Surg 2005 34 464 472 10.1016/j.ijom.2005.04.001 16053863

[B11] Garcia VF Animal bites and Pasturella infections Pediatrics in review 1997 18 127 130 10.1542/pir.18-4-127 9100448

[B12] Goldstein JC Citron DM Wield B Blachman U Sutter VL Miller TA Finegold SM Bacteriology of human and animal bite wounds J. of Clinical microbiology 1978 8 667 672 10.1128/jcm.8.6.667-672.1978 PMC275321 744798

